# Innovative logic “AND” gate gene circuits for bladder cancer treatment: preclinical study

**DOI:** 10.1097/JS9.0000000000002270

**Published:** 2025-02-04

**Authors:** Chaojie Xu, Ying Dong, Dongchen Pei, Xintao Zhang, Xiaohong Han, Congcong Cao, Baorui Wu, Changning Lv, Zhengjun Kang, Liqun Zhou, Yuchen Liu, Lin Yao

**Affiliations:** a Department of Urology, Peking University First Hospital, Peking University, Beijing, China; b Shenzhen Institute of Translational Medicine, Shenzhen Second People’s Hospital, The First Affiliated Hospital of Shenzhen University, Health Science Center, Shenzhen University, Shenzhen, China; c Shenzhen Institute of Synthetic Biology, Shenzhen Institutes of Advanced Technology, Chinese Academy of Sciences, Shenzhen, China; d Department of Urology, The First Affiliated Hospital of Shenzhen University, Shenzhen Second People’s Hospital, Shenzhen, China; e Department of Urology, the Fifth Affiliated Hospital of Zhengzhou University, Zhengzhou University, Zhengzhou, Henan Province, China; f Department of Urology, The First Affiliated Hospital of USTC, Division of Life Sciences and Medicine, University of Science and Technology of China, Hefei, China

**Keywords:** bladder cancer, cancer cell signaling, gene circuit, logic “AND” gate dual-target genetic circuit, synthetic biology

## Abstract

In the evolving field of precision oncology, the synthesis of gene circuits that specifically target cancer cells while preserving normal tissue marks a significant breakthrough. However, traditional approaches typically concentrate on single-gene targets, lacking the directed recognition and control among the intricate networks of signaling pathways. Our study presents a synthetic gene circuit, the Logic “AND” Gate Dual-Target Genetic Circuit (LAG-DTGC), which integrates multiple signals to achieve comprehensive reprogramming of various signaling pathways in bladder cancer (BC) cells. This circuit’s development hinged on detailed bioinformatics analysis, pinpointing more unique biomarkers with similar expression pattern in BC. LAG-DTGC is engineered to selectively activate in cells where these biomarkers are abnormally expressed. Its precision and the remodeling cell behavior capability are further enhanced by incorporating a logic “AND” gate, triggering the circuit only in the presence of these aberrant cancer-specific biomarkers. LAG-DTGC exhibits an extraordinary ability to reprogram cancer cell signaling pathways, turning the cells’ own mechanisms against them for therapeutic effect. This work highlights the potential of synthetic biology in developing precise, less toxic treatments for BC. The LAG-DTGC represents a promising new paradigm in cancer therapy.

## Introduction

**Highlights**

- First to develop a Logic 'AND' Gate Dual-Target Genetic Circuit (LAG-DTGC) specifically for bladder cancer.
- Achieved over 60% apoptosis induction in bladder cancer cells while sparing normal tissues.
- Demonstrated a 70% reduction in tumor growth in preclinical mouse models.
- Pioneering approach with significant potential for clinical translation in targeted bladder cancer therapy.

Bladder cancer (BC) is one of the most aggressive human genitourinary cancers, has the highest published incidence involving malignant urinary system tumors^[1]^. Despite various therapeutic approaches, the clinical outcomes for BC have not improved significantly over the past 30 years, as the 5-year survival rates remain stagnant^[2-4]^. The primary limitations of conventional therapies, such as chemotherapy and radiotherapy, include severe side effects due to their lack of specificity, impacting not only cancer cells but also surrounding healthy tissues. These therapies are often ineffective at the molecular level for metastatic tumors, as they fail to address the complex signaling networks involved in cancer progression. Moreover, although targeted therapies were developed to address these issues by focusing on single-gene targets, they lack the ability to comprehensively modulate cancer’s signaling complexity. By merely targeting isolated molecular components, single-gene approaches often touch only fragments of the tumor’s molecular framework, without the necessary level of control across the complex network of signaling pathways that drive cancer progression. This limitation highlights the need for a strategy that can provide more coordinated regulation across multiple oncogenic pathways, which is especially critical for managing advanced and heterogeneous tumors like BC.

In an effort to transcend these limitations, our research introduces a sophisticated strategy employing multi-logic gate genetic circuits. This innovative approach seeks to orchestrate a meticulous and simultaneous reprogramming of several key oncogenic signaling pathways, thereby potentially establishing a new standard for comprehensive molecular intervention in BC therapy.

The artificial gene circuits are systems composed of multiple genes that can be designed as a controllable signal transduction network^[5]^. Artificial gene circuit brings new capabilities to BC treatments researches. By regulating the internal signal transduction of cells, artificial gene circuits can achieve precise killing of tumor cells, thereby improving treatment efficacy. One of the most commonly used tools in the construction of gene circuit is the CRISPR/Cas9 system, which enables precise editing of DNA sequences^[6]^. However, the conventional form of Cas9 protein is limited to double-stranded DNA breaks, thereby restricting its application in gene regulation. A variant of Cas9 protein, called nuclease-dead mutants of Cas9 (dCas9), which lacks the catalytic activity of Cas9 but can still bind to RNA and form a stable complex with DNA^[7]^ was developed to overcome the limitation.

To further enhance targeting specificity, high-throughput sequencing techniques and bioinformatics analyses are used to identify genes with similar expression patterns^[6]^. By integrating these genes into artificial genetic circuits and modulating downstream oncogenic pathways, it is possible to precisely reprogram cancer signaling networks in BC cells without affecting normal cells, enhancing therapeutic outcomes. In this study, we employed a comprehensive screening strategy that incorporated multiple bioinformatics analysis techniques, such as weighted gene co-expression network analysis (WGCNA), single-factor COX regression analysis, LASSO regression, and multiple-factor COX regression analysis. This strategy was applied to transcriptome data to identify potential BC-specific targets. We constructed a dual-pathway target gene circuit, termed the Logic “AND” Gate Dual-Target Genetic Circuit (LAG-DTGC). Only when the gene has the same expression pattern can the LAG-DTGC be activated to ensure more thorough regulation.

Some genes with similar expression patterns were screened out in our study to involve in the construction of gene circuits, achieving more thorough reprogramming among multiple signaling pathways in BC cells. This innovative approach to cancer treatment exemplifies the potential of a unified, targeted strategy. The LAG-DTGC offers enhanced specificity and effectiveness in cancer treatment as it targets specific molecular mechanisms involved in cancer progression, while leaving normal cells unharmed.

## Methods

### Cell lines and cell culture

The cell lines used in this study were human BC cell lines 5637, UMUC-3, T24, BIU-87, human bladder normal epithelial cell line SV-HUC1, and human kidney epithelial cell line 293T. All cell lines were purchased from the Shanghai Institute of Life Sciences, Chinese Academy of Sciences. Cells were cultured in a humidified incubator at 37°C with 5% CO_2_. 5637, UMUC-3, T24, and BIU-87 cell lines were maintained in RPMI-1640 medium (Gibco) supplemented with 10% fetal bovine serum (FBS, Gibco) and 1% penicillin/streptomycin (Gibco). SV-HUC1 cells were cultured in F-12K medium (Gibco) supplemented with 10% FBS and 1% penicillin/streptomycin. 293T cells were cultured in Dulbecco’s modified Eagle’s medium (DMEM, Gibco) supplemented with 10% FBS and 1% penicillin/streptomycin.

### Data source and preprocessing

Transcriptome data and clinical data of BC patients (TCGA-BLCA) were downloaded from The Cancer Genome Atlas (TCGA) database (https://portal.gdc.cancer.gov/), including 414 BC samples and 19 bladder adjacent tissue samples. After excluding patients with missing clinical information, a total of 407 BC patients were included in this study. For transcriptome preprocessing, high-throughput sequencing data in FPKM format were converted to Transcripts Per Million (TPM) for improved comparability across samples. TPM values were then log-transformed (log2(TPM + 1)) to stabilize variance and normalize the data for downstream analysis. To address potential batch effects, we used the ComBat method in the sva package (R), ensuring data consistency across samples.

### Weighted gene co-expression network analysis

Differentially expressed genes (DEGs) between BC and adjacent tissue were identified using the “limma” package with a screening criterion of FDR < 0.05 and |log2 FC| > 1. WGCNA was performed on all DEGs to identify the gene modules most related to BC development. Firstly, a weighted co-expression network was constructed based on all DEGs. The correlation between sample features and candidate modules was calculated to determine the highly relevant modules with features and further analyze the central genes. An appropriate β value was chosen to generate a scale-free network (scale-free topology index = 0.90). Then, the “dynamic tree cut” algorithm was used to assign similar genes to the same candidate module with a minimum size of 60. Pearson correlation analysis (*P* < 0.05) was used to analyze the correlation between module feature genes and sample features. The most significantly related module to BC development was extracted for further analysis.

### Construction of a prognostic gene signature for bladder cancer development and progression

To explore the prognostic role of genes associated with BC development and progression, the most significant module genes were selected to construct a prognostic risk signature. Firstly, univariate Cox regression analysis was used to identify candidate genes significantly associated with overall survival (*P* < 0.05). To prevent overfitting, the “glmnet” package was used for the least absolute shrinkage and selection operator (LASSO) algorithm, and the best *λ* value was selected based on the minimum cross-validation error in 10-fold cross-validation. Then, a multivariate Cox regression model analysis was performed to identify core genes and calculate their respective coefficients. The risk score of each sample was calculated based on the results of the multivariate Cox regression, using the formula:

$$Riskscore=\overset{n}{\underset{i=1}{\sum }}\left(coefi*Expi\right)$$

where Exp represents the gene expression level, and coef is the regression coefficient in the multivariate Cox regression analysis as described previously. Exp represents the gene expression level.

Using the risk score formula, the risk scores of each TCGA-BLCA sample were calculated. All samples were divided into low-risk and high-risk groups based on the median value of the risk score. Kaplan–Meier survival curves were drawn using the R package “survival” to determine the prognostic differences between the high-risk and low-risk groups. Additionally, receiver operating characteristic (ROC) curve analysis was performed to validate the prognostic value of the risk score. Univariate and multivariate Cox regression analyses were conducted to validate the effectiveness of the risk score as an independent prognostic indicator for clinical variables and risk scores.

### Construction and validation of a clinical prediction model

To identify the best prognostic indicators for overall survival at 1, 3, and 5 years for patients with BC, we conducted ROC analyses of risk scores, age, sex, tumor grade, and clinical-pathologic stage. To quantify the probability of survival at 1, 3, and 5 years for patients, we constructed a nomogram that combined the risk score with other clinical-pathologic features. Calibration curves were used to assess the performance of the model.

### Functional enrichment analysis

We used GSEA 4.1 software to analyze the functional annotation of potential specific target genes in BC using the c5.go.v7.4.symbols collection. Results were considered statistically significant when *P* < 0.05. The top eight outcomes were selected for visualization.

### Immunohistochemistry staining procedure

Immunohistochemistry staining was conducted on tissue microarray (TMA) slides (HBlaU060CS02, Shanghai Outdo Biotech Company, China) using CNTN1 (13843-1-AP, Proteintech) and COL14A1 (AP22329c, Abcepta) antibodies. The TMA slides were deparaffinized to water, and antigen retrieval was performed. The slides were then treated with 3% hydrogen peroxide to block endogenous peroxidase activity. After circling the sections with a hydrophobic pen, they were blocked with serum. Primary antibodies against CNTN1 (1:800) and COL14A1 (1:800) were incubated overnight at 4°C. The next day, the slides were incubated with a secondary antibody at room temperature, followed by color development with DAB. The nuclei were counterstained with hematoxylin, and the slides were dehydrated, cleared, and mounted. The immunoreactive score (IRS) was calculated as the product of staining intensity (SI) and the percentage of positive cells (PP). The SI was graded on a scale of 0 to 3 (0: no staining, 1: weak, 2: moderate, 3: strong), and the PP was categorized into five levels (0: 0–5%, 1: 6–25%, 2: 26–50%, 3: 51–75%, 4: >75%).

### Construction of a LAG-DTGC

The LAG-DTGC was constructed using two plasmids, each tailored to monitor specific signaling pathways and enable precise gene activation under dual conditions (Supplemental Digital Content Table 1, http://links.lww.com/JS9/D862).

Plasmid A was based on the CV130 backbone. To construct Plasmid A, we synthesized an artificial promoter that responds to TGF-β/Smad pathway activity and inserted it upstream of the dCas9-VPR fusion gene. The dCas9-VPR sequence was obtained via PCR amplification, adding PacI and EcoRI restriction sites through specific primers. This fragment was then digested with PacI and EcoRI and ligated into the CV130 vector at the corresponding sites using T4 DNA ligase. Following ligation, the plasmid was transformed into competent *E. coli* cells and cultured on selective media, where positive colonies were isolated for further verification.

Plasmid B was constructed using the CV268 vector. This plasmid included a second artificial promoter that activates transcription only upon MAPK/ERK pathway activation. A ribozyme-sgRNA-ribozyme sequence targeting the BAX gene was synthesized and amplified by PCR with PacI and NheI restriction sites for cloning into the PacI/NheI site of the CV268 vector. After ligation, the plasmid was transformed into competent cells and screened on selective media to confirm successful insertion.

To verify the construction, colony PCR and Sanger sequencing were performed with specific primer sets (Supplemental Digital Content Table 2, http://links.lww.com/JS9/D862). Functionality was assessed by co-transfecting the plasmids into BC cells, with subsequent qPCR and Western blot analysis confirming BAX gene activation exclusively under conditions where both TGF-β/Smad and MAPK/ERK pathways were active, ensuring that only cancer cells expressing these signals would activate the gene circuit while sparing normal cells.

### Lentiviral packaging of LAG-DTGC

The lentiviral vectors for overexpressing gene circuits A and B were constructed with the assistance of Shanghai Jikai Gene Chemical Technology. HEK293T cells, utilized for lentivirus packaging, were seeded at a density of 3 × 10^5 cells per well in 12-well plates and incubated for 24 hours. Following incubation, the original culture medium was aspirated and replaced with fresh complete medium containing calculated viral titers, transfection reagent, and viruses. This mixture was gently homogenized before being reintroduced to the cells, which were subsequently returned to the incubator. Observation under a microscope was conducted between 6 and 12 hours post-transfection to assess cell morphology and health, prompting medium replacement based on cell condition. At 72 hours post-infection, puromycin was added to commence selection of stably transduced cells, continuing for 1–2 weeks. The surviving cells represented the stably transduced populations, suitable for subsequent experimental use.

### AAV packaging of LAG-DTGC

For the AAV packaging of gene circuits A and B, the same HEK293T cells were transfected using polyethylenimine (PEI) alongside appropriate plasmids. Following transfection, the cells were incubated to facilitate viral production. Viral particles were harvested from the culture medium 3–7 days post-transfection. This involved collecting the medium followed by its centrifugation and subsequent purification using gradient ultracentrifugation techniques to obtain high-titer, replication-deficient AAV particles. The produced AAV was then prepared for further experimental applications, particularly for assessing the functionality of the synthetic gene circuits in targeting and modulating oncogenic signaling pathways within BC cells.

### Luciferase assay

To confirm the function of the artificial promoter, a luciferase reporter gene was constructed. First, the artificial promoter was cloned into the GV238 vector upstream of the luciferase reporter gene. The overexpression plasmid for CNTN1 and the plasmid for monitoring TGF-β/Smad pathway activity were co-transfected into 293 T cells, while the overexpression plasmid for COL14A1 and the plasmid for monitoring MAPK/ERK pathway activity were co-transfected into 293 T cells. After 48 hours, the cells were lysed, and the luciferase activity was measured using the Dual-Luciferase Reporter Assay System.

### Western blot analysis

Cells were harvested and lysed using RIPA buffer. The protein concentration was measured using the BCA method. Equal amounts of protein were separated by SDS-PAGE and transferred to PVDF membranes. The membranes were then blocked with 5% non-fat milk and incubated with primary antibodies specific to CNTN1, COL14A1, BAX, and β-actin. After washing, the membranes were incubated with HRP-conjugated secondary antibodies, and the protein bands were visualized using ECL reagents. The experimental strip picture was generated by ChemiCapture software, and the gray value of the measured band was analyzed by ImageJ software, and band intensities (gray values) were quantified with ImageJ software. For statistical analysis, gray values of protein bands from three independent experiments were quantified and normalized to β-actin, used as an internal control. The mean values and standard deviations (SD) were calculated, and differences between experimental and control groups were evaluated using an unpaired Student’s t-test. *P* < 0.05 were considered statistically significant, and results were visualized using GraphPad Prism.

### Cell apoptosis assay

Apoptosis was measured by flow cytometry using Annexin V-FITC/PI double staining. Stable cell lines (5637, T24, and SV-HUC1) carrying the LAG-DTGC, along with control cells, were seeded in 6-well plates. After 48 hours, cells were collected, washed with cold PBS, and resuspended in binding buffer. They were then incubated with Annexin V-FITC and PI, followed by flow cytometric analysis. For apoptosis data analysis, cells were classified into four quadrants (live, early apoptotic, late apoptotic, and necrotic) using FlowJo software. The percentages of apoptotic cells (early and late apoptosis) were averaged across three independent experiments. Differences between experimental and control groups were assessed using Student’s t-test, with *P* < 0.05 considered statistically significant. GraphPad Prism was used for visualizing the data.

### Cell viability assay

Cell viability was measured using the CCK-8 assay. After transfection, cells were seeded in 96-well plates and incubated for 0, 12, 24, and 48 hours. Then, 10 μL of CCK-8 reagent was added to each well, and the plates were incubated for an additional 2 hours. The absorbance was measured at 450 nm using a microplate reader.

### 5-Ethynyl-2ʹ-deoxyuridine (EdU) Incorporation Assay

Cell proliferation was measured using the EdU assay. After transfection with the LAG-DTGC, cells were seeded in 6-well plates and incubated for 24 hours. Then, 1 ml of EdU was added to each well, and the plates were incubated for an additional 2 hours. The cells were then fixed and stained with Apollo fluorescent dye, and the EdU-positive cells were counted using a fluorescence microscope.

### Wound healing assay

To investigate the effects of the LAG-DTGC on the migration ability of BC cells and normal bladder cells, a wound healing assay was performed. Cells were seeded in 6-well plates at a density of 1 × 10^6^ cells/well and cultured in DMEM medium at 37°C, 5% CO_2_. After reaching 80–90% confluence, a 10 μL sterile pipette tip was used to scratch the cell monolayer. The cells were washed with PBS to remove debris and cultured in serum-free medium for 24 hours. The images of the scratched area were captured at 0, 24, and 48 h after scratching using a microscope (Leica, Germany). Each experiment was performed in triplicate, and the results were expressed as the mean ± standard deviation (SD).

### Transwell invasion assay

Cells were seeded into the upper chamber of a Transwell plate (Corning, USA) coated with Matrigel, and the lower chamber was filled with DMEM medium containing 20% FBS as a chemoattractant. After incubating for 24 hours, the cells that invaded the lower chamber were fixed with 4% paraformaldehyde, stained with 0.1% crystal violet, and observed and counted under a microscope (Leica, Germany). Each experiment was performed in triplicate, and the results were expressed as the mean ± SD.

### Tumor xenograft model

The procedure of the tumor xenograft test was approved by Shenzhen Topo Bioethics Committee (TOP-IACUC-2023-0038)^[8,9]^. Mice were housed under standard laboratory conditions. BALB/c nude mice were randomly assigned to experimental or control groups (six mice per group). BC T24 cells carrying the LAG-DTGC tool (5 × 10^6^) were injected subcutaneously in the experimental group and BC T24 cells carrying the null load (5 × 10^6^) were injected subcutaneously in the control group. Tumor volumes were measured twice a week for three weeks. Tumor volume was calculated using the formula: *V* = *L* × *W*^2^/2, where *L* is the length of the tumor and *W* is the width of the tumor. At the end of the experiment, mice were euthanized, tumors were excised, and peripheral blood was collected.

### Experimental metastatic mouse model

Male B-NDG mice, 5 weeks old, were used in this experiment. These mice were injected with 200 μL of phosphate-buffered saline containing 1 × 10^5^ T24 BC cells that had been genetically modified to express luciferase. After 4 weeks, the mice underwent anesthesia with isoflurane and received intraperitoneal injections of D-luciferin sodium salt at a dosage of 150 mg/kg. The spread of the BC cells was then measured using the Xenogen IVIS system from PerkinElmer, based in Boston, MA, USA. Photon flux from the lung area was quantified using the Living Image 4.3.1 software, also from PerkinElmer/Caliper.

### Quantification of cytokines by ELISA

To quantify cytokines IL-1, IL-6, IL-8, and TNF-α, serum was separated from blood by centrifugation and stored at −80°C. Specific ELISA kits for each cytokine were used. Serum and standards were added to a 96-well plate and incubated for 2 hours at room temperature to allow antigen-antibody binding. After washing the wells to remove unbound substances, enzyme-linked antibodies were added, followed by a substrate solution that reacts to produce a color change. The reaction was stopped, and the optical density was measured at 450 nm. Cytokine concentrations were determined by comparing absorbance values to a standard curve, providing quantitative analysis of cytokine levels in relation to tumor progression.

### Statistical analysis

Between-group differences were analyzed using chi-square tests, Fisher’s exact test, Student’s *t*-test, or analysis of variance (ANOVA). Kaplan–Meier analysis was used for survival analysis, and the log-rank test was used to compare survival differences. ROC curves and calibration curves were used to evaluate model performance. The cut-off points in the Kaplan–Meier curves were determined based on the median risk scores, and the thresholds in the regression models were selected using cross-validation to ensure model stability and accuracy. All data were obtained from at least three independent replicates. A two-tailed *P*-value less than 0.05 was considered to indicate statistical significance. All statistical analyses were performed using R software (version 4.0.5) and GraphPad Prism 7.0. The R packages used included “limma,” “WGCNA,” “regplot,” “glmnet,” “GSVA,” “survival,” and “rms.”

## Results

### Identifying potential bladder cancer-specific targets through predictive gene signature

#### Basic information and differential gene analysis of cases

**Figure 1.**
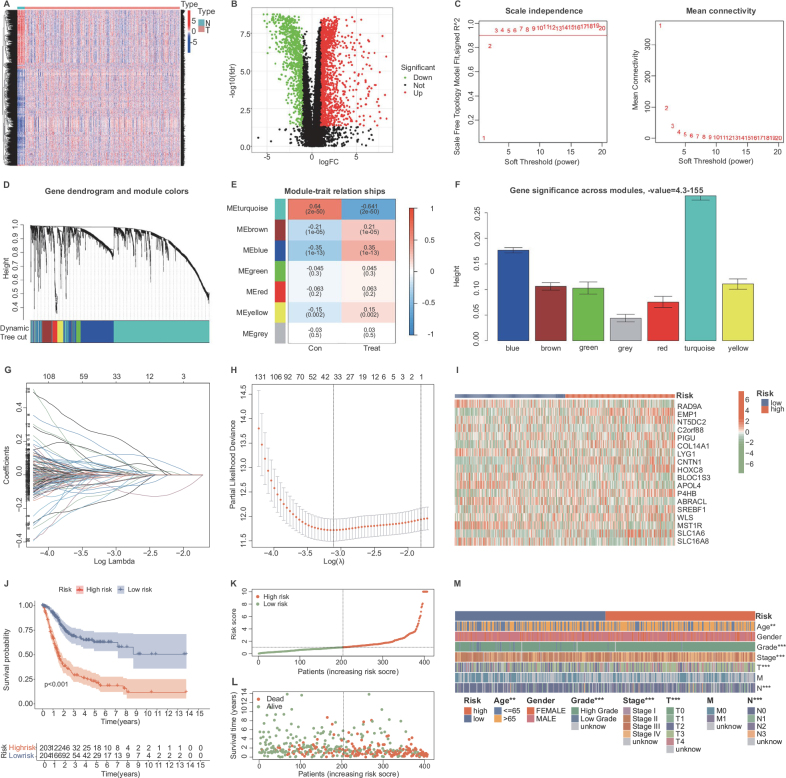
Comprehensive analysis of gene expression and clinical characteristics in bladder cancer patients. (A) Heatmap of differentially expressed genes between bladder cancer and adjacent normal tissues; blue indicates low expression, red indicates high expression. (B) Volcano plot showing upregulated (red) and downregulated (green) genes, with non-significant genes in black. (C) Plot of scale independence vs. soft-threshold power to confirm scale-free network topology. (D) Gene dendrogram with color-coded modules grouping genes by similar expression patterns. (E) Heatmap of correlations between gene modules and clinical features, with color intensity representing correlation strength and direction. (F) Bar plot showing significance (log10(*P*-value)) of genes within each module. (G) Lasso regression plot indicating changes in regression coefficients with increasing log lambda penalty. (H) Cross-validation for Lasso regression, selecting optimal lambda for lowest error. (I) Heatmap comparing gene expression in high-risk and low-risk groups as per prognostic model. (J) Kaplan–Meier survival curves for high-risk (red) vs. low-risk (blue) groups. (K) Distribution of patient risk scores, with high and low-risk thresholds highlighted. (L) Scatter plot of survival status (green = alive, red = deceased) across increasing risk scores. (M) Heatmap showing clinical characteristics and expression of key genes by patient risk score, integrating molecular and clinical data to predict outcomes.

The TCGA-BLCA cohort included a total of 433 samples, including 19 adjacent tissue samples and 414 BC samples. After excluding patients with missing clinical data, a total of 407 BC patients were finally included. As shown in Table 1, the collected basic information of cases included variables such as age, gender, pathological grade, TNM stage, and others. The proportion of elderly patients (60.69%), female patients (73.46%), high pathological grade (94.10%), and TNM advanced stage cases was relatively high. Among them, 177 patients experienced the study endpoint death event, and the median follow-up time of the cohort was 1.45 years. As shown in the heatmap (Fig. 1A) and volcano plot (Fig. 1B), a total of 2656 genes (FDR < 0.05 and |log2FC| > 1) were found to have significant expression differences between BC tissues and adjacent tissues.

**Table 1:** Clinical pathological characteristics of bladder cancer patients in the TCGA database

Characteristics	TCGA-BLCA cohort
	*N* = 407
Age	
≤65	160 (39.31%)
>65	247 (60.69%)
Gender	
Female	108 (26.54%)
Male	299 (73.46%)
Grade	
High	383 (94.10%)
Low	20 (4.92%)
Unknown	4 (0.98%)
Stage	
I-II	131 (32.19%)
III-IV	273 (67.07%)
Unknown	3 (0.74%)
T	
T0-T2	122 (29.98%)
T3-T4	251 (61.67%)
Unknown	34 (8.35%)
M	
M0	195 (47.91%)
M1	11 (2.70%)
unknown	201(49.39%)
N	
N0-N1	282 (69.29%)
N2-N3	82 (20.15%)
Unknown	43 (10.56%)

#### Identification of the most correlated module with bladder cancer occurrence based on WGCNA

A co-expression network was established based on the expression matrix of 2656 genes and the phenotype of BC using the WGCNA to identify the most correlated module with BC occurrence. Firstly, an unweighted network was constructed, and the optimal soft threshold β was set to 9 when the scale-free topology index reached 0.90 (Fig. 1C). The dynamic cutting algorithm was then used to introduce genes with similar expression patterns into the same module (module size = 60) to form a hierarchical clustering tree of different modules. Hierarchical clustering analysis was performed based on weighted correlation, and the clustering results were divided according to the set standards, resulting in seven gene modules (Fig. 1D). Among the seven gene modules, the modules positively correlated with the occurrence and development of BC include the brown, blue, green, red, yellow, and grey modules. However, the turquoise module (cor = − 0.64, *P* = 2e-50) was negatively correlated with BC occurrence and development (Fig. 1E). We focused mainly on the module with the highest correlation with BC occurrence and development; thus, we extracted genes from the turquoise module for further analysis. The gene importance scores of the seven modules are shown in Figure 1F.

#### Construction of a gene signature related to the occurrence and development of bladder cancer

To further investigate the prognostic value of the candidate genes, we extracted the expression matrix and follow-up information of 1531 genes from the turquoise module in the TCGA-BLCA cohort. First, a univariate Cox regression analysis was performed, and 477 genes were identified as having significant prognostic value (*P* < 0.05, Supplemental Digital Content Table 3, http://links.lww.com/JS9/D862). To avoid overfitting, these genes were subjected to Lasso regression analysis (Fig. 1G), and the optimal value of the penalty coefficient *λ* was determined by 10-fold cross-validation (Fig. 1H). Finally, a multivariate Cox regression analysis was performed to further screen and select 18 genes to construct a prognostic model for BC occurrence and development, including RAD9A, EMP1, NT5DC2, C2orf88, PIGU, COL14A1, LYG1, CNTN1, HOXC8, BLOC1S3, APOL4, P4HB, ABRACL, SREBF1, WLS, MST1R, SLC1A6, and SLC16A8. These genes are considered to be potential BC-specific targets. By analyzing the gene expression levels of the 18 genes in the TCGA database (Fig. 1I), we found that most genes have abnormal expression patterns in BC tissues compared to normal tissues.

These 18 genes were included in the formula for calculating the risk score (Table 2, section 2.4) to obtain the risk score for each patient. Based on the median risk score (1.035), patients in the TCGA-BLCA cohort were divided into high-risk and low-risk subgroups. Kaplan–Meier survival curves showed that the overall survival time of patients in the high-risk group was significantly shorter than that in the low-risk group (*P* < 0.001; Fig. 1J). In addition, the distribution of patient survival status and risk scores indicated that low-risk patients had a longer overall survival time (Fig. 1K-L). Figure 1M displays a heatmap correlating patient risk groups with gene expression and clinical data in the TCGA-BLCA cohort. Columns, ordered by risk score, show gene expression levels for the 18 genes, and patient demographics like age and cancer stage. The visualization highlights the alignment of high-risk scores with more aggressive cancer traits, substantiating the risk model’s effectiveness in predicting outcomes and guiding personalized treatment strategies in BC.

**Table 2:** Gene regression coefficients for building clinical prediction models

ID	Coeff
RAD9A	−0.259589647022857
EMP1	0.121426431489606
NT5DC2	0.295606382448671
C2orf88	−0.359726786173335
PIGU	0.360586452439354
COL14A1	0.106181046057204
LYG1	−0.195207161944943
CNTN1	0.129192115067675
HOXC8	0.267527333235735
BLOC1S3	−0.359939090857547
APOL4	−0.145671327472594
P4HB	0.349414027177053
ABRACL	−0.259820442011449
SREBF1	0.132236942516663
WLS	0.129336219986891
MST1R	−0.129521554582859
SLC1A6	0.117052917080282
SLC16A8	−0.311487652336096

**Figure 2.**
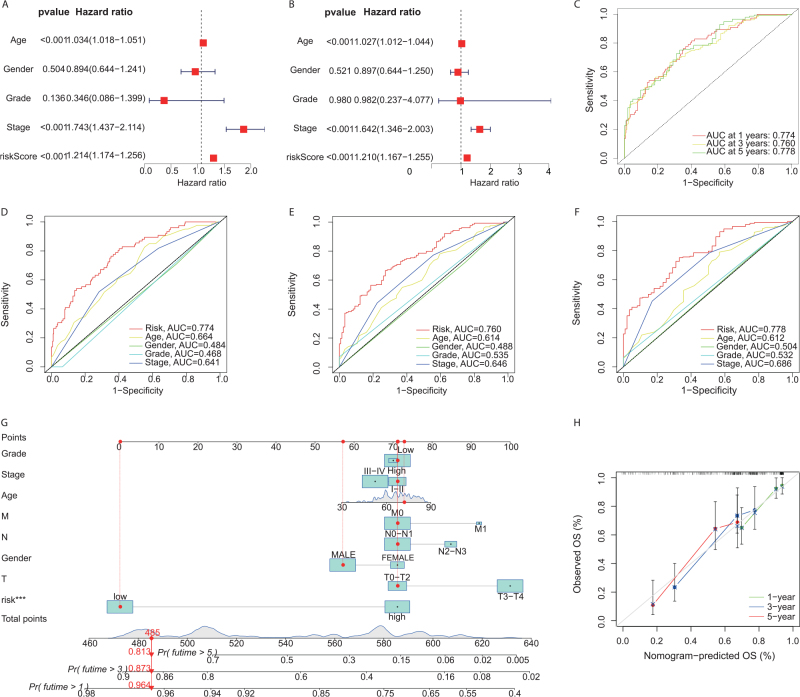
Development and validation of a clinical prediction model for bladder cancer. (A-B) Univariate and multivariate cox regression analyses identify significant prognostic factors (age, gender, grade, stage, risk score) influencing bladder cancer outcomes, with hazard ratios and *P*-values depicted. (C-F) Receiver operating characteristic (ROC) curves evaluate the predictive accuracy of the risk score for 1-year (D), 3-year (E), and 5-year (F) survival, highlighting the model’s superior performance with area under curve (AUC) metrics. (G) A Nomogram integrates multiple factors to estimate survival probabilities at 1, 3, and 5 years, assigning points to each prognostic factor. (H) Calibration curves assess the nomogram’s predictive accuracy by comparing nomogram-predicted versus observed survival rates, ensuring model reliability across different time frames.

To validate whether the risk score can serve as an independent risk factor affecting the prognosis of BC patients, we included the patients’ clinical information (age, sex, pathological grade, TNM stage, and risk score) for Cox regression analysis. The univariate Cox analysis showed that the hazard ratio (HR) of the risk score was 1.214 (95%CI: 1.174–1.256; *P* < 0.001; Fig. 2A), indicating a statistically significant difference. To correct for the effects of other factors, multivariate Cox regression analysis showed that the HR of the risk score was 1.210 (95%CI: 1.167–1.255; *P* < 0.001; Fig. 2B). Subsequently, we plotted the ROC curves, and the AUC values for the 1-, 3-, and 5-year overall survival of BC patients were 0.774, 0.760, and 0.778, respectively, indicating a good discrimination effect of the prognosis (Fig. 2C). To further validate that the risk score is the best prognostic indicator among multiple clinical variables, age, sex, clinical stage, and tumor grade were included as candidate variables in the model. The results of the AUC for the 1-year, 3-year, and 5-year overall survival curves indicated that the risk score obtained the highest AUC value (Fig. 2D-F). Taken together, these results support that the risk score can serve as an independent prognostic indicator for BC. Our 18-gene signature has good predictive ability for clinical outcomes.

#### Construction and performance evaluation of a clinical prediction model

We subsequently developed a nomogram consisting of a risk score and clinical variables (gender, age, tumor grade, TNM stage) to quantitatively predict the 1-year, 3-year, and 5-year survival probabilities of BC patients (Fig. 2G). For example, for a 72-year-old male patient diagnosed with BC, T1N0M0, high tumor grade, and low-risk group, the nomogram score was calculated as 485, indicating a total survival rate of 96.4%, 87.3%, and 81.3% at 1, 3, and 5 years, respectively. The calibration curve demonstrated good calibration of the developed model (Fig. 2H).

### Innovative dual-target gene circuit design: regulating oncogenic signaling pathways

#### Identification of potential downstream regulatory pathways for bladder cancer targets CNTN1 and COL14A1

**Figure 3.**
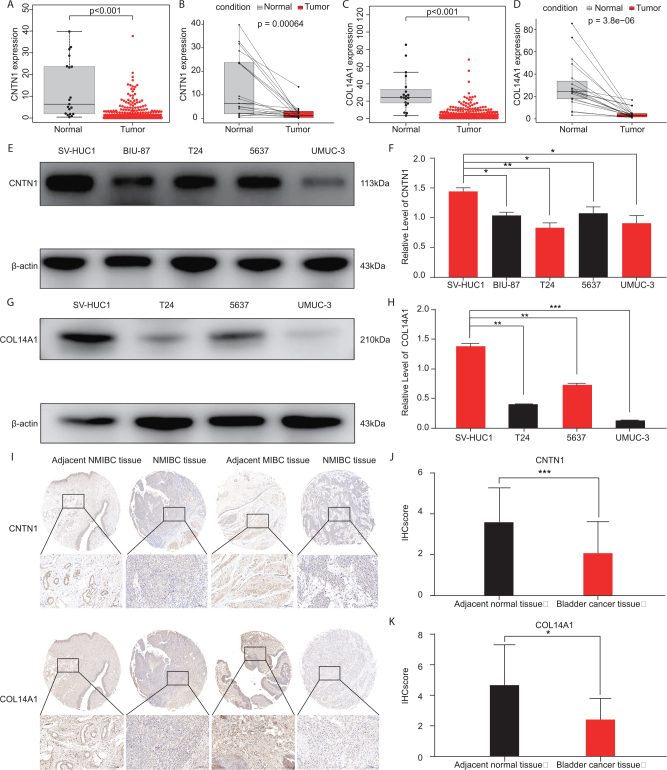
Expression patterns of genes CNTN1 and COL14A1 in bladder cancer tissue and adjacent tissue. (A) Box plots showing CNTN1 expression levels in bladder cancer tissue versus adjacent normal tissue. (B) Paired comparison of CNTN1 expression levels between matched normal and tumor tissues from the same patients. (C) Box plots showing COL14A1 expression levels in bladder cancer tissue versus adjacent normal tissue. (D) Paired comparison of COL14A1 expression levels between matched normal and tumor tissues from the same patients. (E, F) Western blot analysis of CNTN1 protein levels in five cell lines: SV-HUC1, BIU-87, T24, 5637, and UMUC3. (G, H) Western blot analysis of COL14A1 protein levels in four cell lines: SV-HUC1, T24, 5637, and UMUC3. (I) Representative immunohistochemistry staining images for CNTN1 and COL14A1 in adjacent normal bladder mucosa and bladder cancer tissues. (J) Histogram of immunohistochemistry scores (H-scores) for CNTN1 in adjacent normal tissues and bladder cancer tissues. (K) Histogram of immunohistochemistry scores (H-scores) for COL14A1 in adjacent normal tissues and bladder cancer tissues. Error bars indicate mean ± standard deviation. Each experiment was repeated at least three times. Statistical significance is marked as follows: ^ns^*P* > 0.05, *P < 0.05, **P < 0.01, ***P < 0.001.

To test the specificity of the potential 18 BC targets identified, we selected two targets (CNTN1 and COL14A1) for validation. Firstly, we examined the expression levels of CNTN1 and COL14A1 genes using data from TCGA database and found that their expression levels were significantly reduced in BC tissues compared to normal tissues (Fig. 3A, C). Furthermore, paired analysis of CNTN1 and COL14A1 expression levels in matched normal and tumor tissues from the same patients showed a significant reduction in tumor tissues (Fig. 3B, D). Subsequently, we validated these findings in cell lines, and found that the protein expression levels of CNTN1 and COL14A1 were significantly lower in BC epithelial cell lines (5637, UMUC-3, T24, BIU-87) compared to normal bladder epithelial cell line (SV-HUC1) (Fig. 3E-H). To further validate these findings, we used tissue microarray (TMA) slides containing BC tissues and adjacent normal bladder mucosa from 30 patients. These TMAs included a range of BC stages according to the AJCC seventh edition staging system (Ta-T2: 11 cases, T3-T4: 19 cases), as well as patient demographics, providing a comprehensive representation of BC pathology. Specifically, the TMAs included 22 cases of muscle-invasive BC (MIBC) and 8 cases of non-muscle-invasive BC (NMIBC). The TMA slides were stained using immunohistochemistry (IHC) with specific antibodies against CNTN1 and COL14A1. Representative images of IHC staining showed that both CNTN1 and COL14A1 were strongly expressed in adjacent normal bladder mucosa, while their expression was markedly reduced in BC tissues (Fig. 3I). Quantitative analysis of the immunohistochemistry scores (H-scores) confirmed that the expression levels of CNTN1 and COL14A1 were significantly lower in BC tissues compared to adjacent normal tissues (Fig. 3J, K). These results suggest that CNTN1 and COL14A1 may be potential targets for BC.

**Figure 4.**
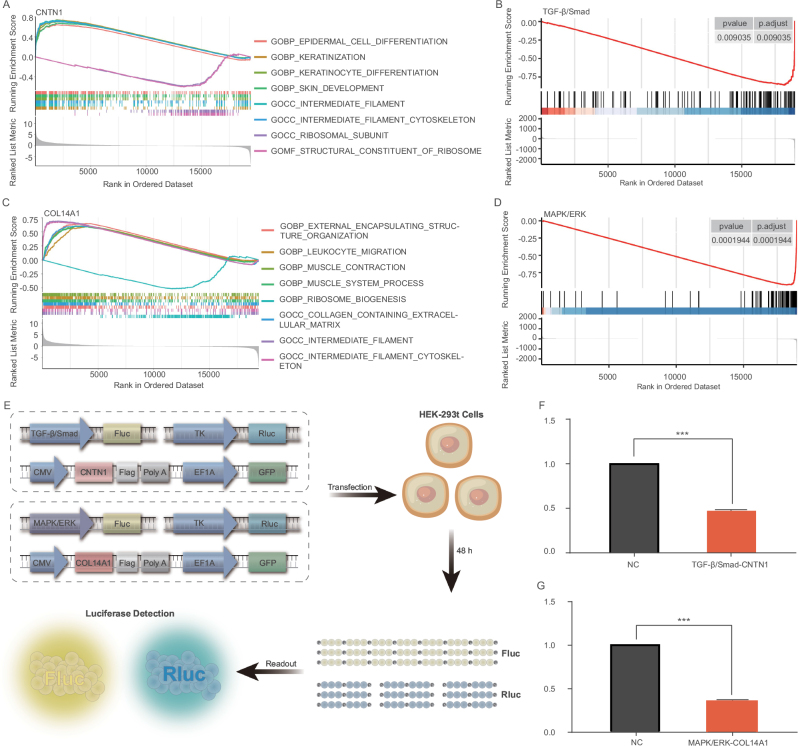
Functional analysis and signaling pathway assessment of CNTN1 and COL14A1 in HEK-293T cells. (A) Gene ontology (GO) enrichment analysis for CNTN1, highlighting associated processes such as epidermal cell differentiation, keratinization, and other skin-related functions. (B) Enrichment plot for TGF-β/Smad signaling influenced by CNTN1, with significant peaks indicating pathway activation. (C) GO enrichment analysis for COL14A1, identifying key processes related to extracellular matrix organization, leukocyte migration, and muscle system activities. (D) Enrichment plot for MAPK/ERK signaling influenced by COL14A1, showing peaks that indicate pathway involvement. (E) Schematic of the dual-luciferase reporter assay system used to assess pathway activity, with constructs for TGF-β/Smad and MAPK/ERK pathways linked to CNTN1 and COL14A1, respectively, in HEK-293T cells. (F-G) Luciferase reporter assay results post-transfection, showing the effects of CNTN1 and COL14A1 overexpression on TGF-β/Smad and MAPK/ERK pathways. Graphs display relative luciferase activities, with statistical significance denoted as follows: ns (not significant), *P < 0.05, **P < 0.01, ***P < 0.001.

To explore the biological roles of CNTN1 and COL14A1, we performed a series of gene expression and functional enrichment analyses. Dividing the samples into high and low expression groups based on the median expressions of CNTN1 and COL14A1, Gene Set Enrichment Analysis (GSEA) revealed significant associations. High expression levels of CNTN1 predominantly correlated with gene sets involved in cell adhesion pathways, indicating its potential role in modulating cellular attachment and migration capabilities in BC (Fig. 4A). Additionally, the TGF-β/Smad pathway analysis revealed significant downregulation, indicating CNTN1’s inhibitory effect on this signaling pathway (Fig. 4B). Similarly, high expression of COL14A1 was linked to pathways crucial for cell migration and adhesion, underscoring its possible function in cancer cell dynamics (Fig. 4C). The MAPK/ERK pathway analysis further supported its negative regulatory role, as evidenced by the decrease in pathway activity (Fig. 4D).

Further molecular investigations were conducted to understand the regulatory mechanisms by which CNTN1 and COL14A1 might influence BC progression. The schematic in Figure 4E illustrates the constructs and methodology utilized in the dual-luciferase reporter assays conducted on HEK-293 T cells. For the assay, we utilized specific plasmid constructs containing either the TGF-β/Smad or MAPK/ERK pathway-responsive elements upstream of a firefly luciferase reporter gene (Fluc), which served as the primary reporter to assess pathway activity. Additionally, each construct included a Renilla luciferase gene (Rluc) under a constitutive promoter as an internal control to normalize the data for transfection efficiency and cell viability. The constructs for the TGF-β/Smad pathway assay contained promoter sequences responsive to Smad signaling, linked directly to the firefly luciferase reporter. In the case of COL14A1, the constructs for the MAPK/ERK pathway assay included MAPK/ERK-responsive elements to drive the expression of the firefly luciferase reporter. These reporter constructs were co-transfected into HEK-293 T cells along with expression vectors for CNTN1 or COL14A1, tagged with a CMV promoter for high expression. Post-transfection (48 hours), cells were lysed and the luciferase activity was measured using a dual-luciferase reporter assay system. Dual-luciferase reporter assays in HEK-293T cells demonstrated that CNTN1 negatively regulates the TGF-β/Smad pathway, evidenced by a fold change of 0.432 in luciferase activity, suggesting a suppressive interaction with this pathway (Fig. 4F). Concurrently, COL14A1 exhibited a negative regulatory effect on the MAPK/ERK pathway with a fold change of 0.416, pointing to its potential inhibitory role in this signaling cascade (Fig. 4G).

#### Construction of a dual-target gene genetic circuit using a logical “AND” gate

**Figure 5.**
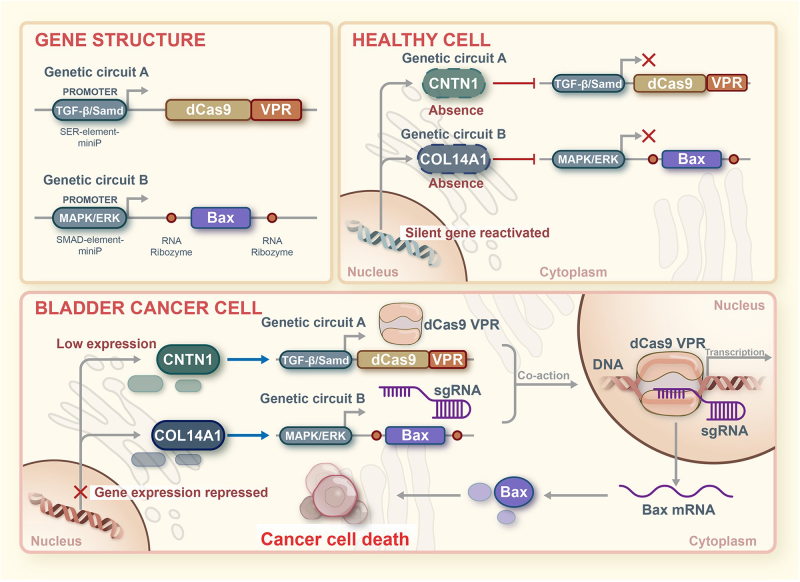
Schematic diagram of the construction mechanism of the logic “AND” gate gene circuit. This figure illustrates the design and functional mechanism of a synthetic gene circuit employing a logic “AND” gate, specifically engineered to target bladder cancer cells. **Gene structure**: The diagram details two distinct genetic circuits: Genetic Circuit A, equipped with a TGF-β/Smad-responsive promoter controlling the expression of a deactivated Cas9 (dCas9) fused to the VP64-p65-Rta (VPR) transcriptional activation complex, designed to activate gene expression upon specific TGF-β/Smad pathway activation; and genetic circuit B, which features a MAPK/ERK-responsive promoter linked to the expression of the pro-apoptotic gene Bax, activated only in the presence of MAPK/ERK pathway signals. **Healthy cell**: In healthy cells, CNTN1 and COL14A1 expression levels are adequate to inhibit the activation of Genetic Circuit A and B, ensuring no Bax expression or effect on normal cellular function. **Bladder cancer cell**: In bladder cancer cells, low expression of CNTN1 and COL14A1 releases the inhibition, leading to the activation of both circuits—CNTN1 activating TGF-β/Smad and COL14A1 activating MAPK/ERK pathways—resulting in the targeted expression of the Bax gene, which promotes cancer cell apoptosis. This therapeutic strategy leverages specific molecular changes within cancer cells, differentiating between healthy and malignant cells.

Based on the BC targets CNTN1 and COL14A1, we designed and constructed a LAG-DTGC. The LAG-DTGC can reprogram the cell carcinogenic signaling pathway, control the expression of the apoptosis gene BAX, and achieve specific intervention to kill BC cells without affecting normal cells. The designed LAG-DTGC used the CRISPR-dCas9 system, functional units (transcriptional activators), an artificial promoter that monitors TGF-β/Smad pathway activity, an artificial promoter that monitors MAPK/ERK pathway activity, ribozyme, and sgRNA (BAX) to construct two parts, genetic circuit A and genetic circuit B, as shown in Figure 5. The specific sequences are provided in Supplemental Digital Content Table 4, http://links.lww.com/JS9/D862.

In BC cells, the downstream regulatory pathways (TGF-β/Smad and MAPK/ERK) were activated due to the low expression of CNTN1 and COL14A1. genetic circuit A started with an artificial promoter that monitored TGF-β/Smad pathway activity and drove the transcription of dCas9 protein and functional units (transcriptional activators) mRNA. Genetic circuit B started with an artificial promoter that monitored MAPK/ERK pathway activity and controlled the transcription of sgRNA targeting BAX gene. To achieve efficient expression of sgRNA in the cell nucleus, we connected ribozymes to both sides of the sgRNA. Finally, the output signal of this circuit led to cell apoptosis. Theoretically, in normal cell lines, the transcriptional expression of genetic circuit A and genetic circuit B is low, and the expression of the apoptosis gene BAX is not affected or only slightly affected, which is not enough to change the fate of the cell. Compared to normal cells, BC cells have low expression of CNTN1 and COL14A1, which activate downstream regulatory pathways (TGF-β/Smad and MAPK/ERK) negatively (Supplemental Digital Content Figure S1–4, http://links.lww.com/JS9/D862). Therefore, this designed LAG-DTGC only received effective output signals in BC cells, achieving specific intervention to kill BC cells without affecting normal cells.

**Figure 6.**
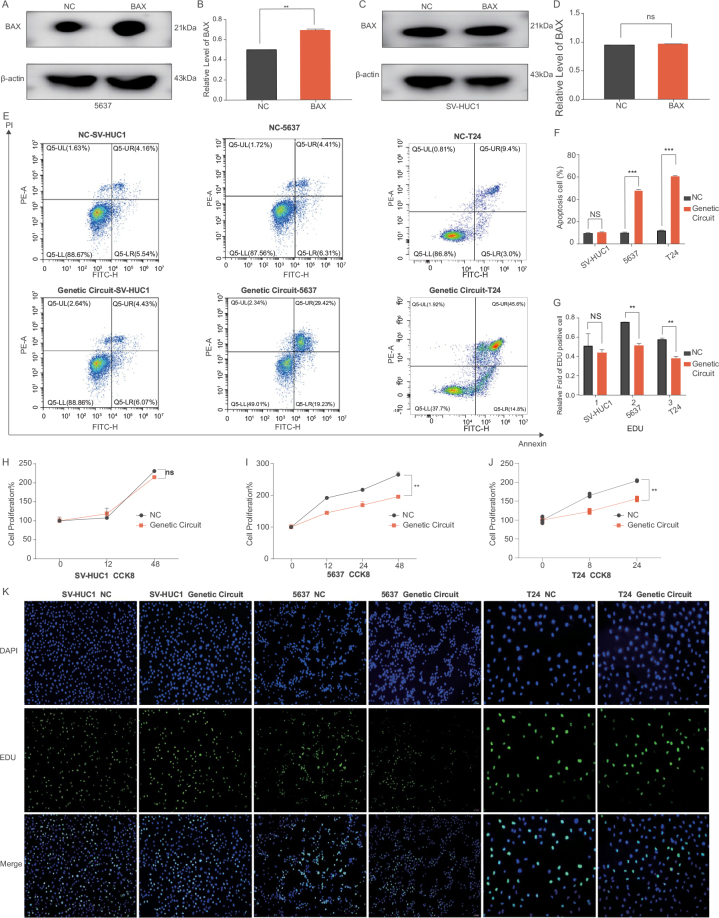
Functional assessment of logic “AND” gate gene circuit in cell lines. (A, B) Construction of stable clones with the logic “AND” gate gene circuit in SV-HUC1 cells via lentiviral infection. Western blot analysis shows no significant difference in BAX protein expression between SV-HUC1 cells with and without the gene circuit. (C, D) Construction of stable clones with the logic “AND” gate gene circuit in 5637 cells. Western blot analysis shows significantly higher BAX protein expression in 5637 cells carrying the gene circuit compared to controls. (E, F) Flow cytometry analysis of apoptosis in SV-HUC1, 5637, and T24 cells. No significant difference in apoptosis rates was observed in SV-HUC1 cells with and without the gene circuit, while apoptosis rates were significantly higher in 5637 and T24 cells with the gene circuit compared to controls. (G, K) EdU proliferation assays comparing SV-HUC1, 5637, and T24 cells with and without the gene circuit. SV-HUC1 cells showed no significant differences in proliferation, while 5637 and T24 cells with the gene circuit exhibited a significant reduction in proliferation compared to controls. (H, I, J) CCK-8 proliferation assays corroborate the EdU results, with no difference observed in SV-HUC1 cells but significantly inhibited proliferation in 5637 and T24 cells carrying the gene circuit compared to controls. Each experiment was performed at least three times. Statistical significance is indicated as follows: ns (*P* > 0.05), *P < 0.05, **P< 0.01, ***P < 0.001.

Next, we verified whether this tool could function normally in cells by constructing stable cell lines of the BC and normal cell lines with LAG-DTGC. The Western blot results showed that the output signal BAX had a higher translation efficiency in the BC cell line (Fig. 6A-B), while the expression of the apoptosis gene BAX was not affected in the normal cell line (Fig. 6C-D), confirming our hypothesis (Supplemental Digital Content Figures S5–6, http://links.lww.com/JS9/D862).

### Validating the efficacy of LAG-DTGC: A novel approach for bladder cancer therapy

#### Promotion of apoptosis in bladder cancer cells with LAG-DTGC

To investigate the effects of LAG-DTGC on apoptosis in normal bladder epithelial cells and BC cells, we performed apoptosis double staining experiments using flow cytometry and detected the activity of the apoptosis protein Caspase-3. As shown in Figure 6E-F, the proportion of apoptotic cells in the experimental group of BC cells (5637 and T24) increased significantly compared to the control group (*P* < 0.05), while there was no significant difference in the apoptosis rate between the control and experimental groups in the SV-HUC1 cell line (*P* > 0.05). These results indicate that LAG-DTGC activates apoptosis pathways specifically in BC cells without affecting normal cells. This targeted apoptotic effect suggests that LAG-DTGC could reduce tumor mass in a controlled manner, with a lower likelihood of harming surrounding healthy tissues—an essential consideration in reducing treatment-related toxicity for clinical applications.

#### Inhibition of bladder cancer cell proliferation with LAG-DTGC

We used CCK-8 and EdU assays to assess the effects of LAG-DTGC on the proliferation of normal bladder cells (SV-HUC1) and BC cells (5637 and T24). CCK-8 data (Fig. 6H-J) showed that the number of BC cells in the experimental group was significantly lower than in the control group (*P*<0.05), with no significant change observed in the normal cell line (*P* > 0.05). Similarly, EdU results (Fig. 6K, G) confirmed that LAG-DTGC significantly inhibited proliferation in BC cells, with no substantial effect on normal cells (*P* > 0.05). The selective inhibition of cancer cell proliferation suggests that LAG-DTGC disrupts key proliferative pathways in cancer cells. By specifically targeting these pathways, LAG-DTGC could serve as a therapeutic tool to control tumor growth with minimal impact on normal cell function, potentially improving therapeutic precision and reducing side effects in a clinical setting.

#### Specific inhibition of bladder cancer cell migration and invasion with LAG-DTGC

**Figure 7.**
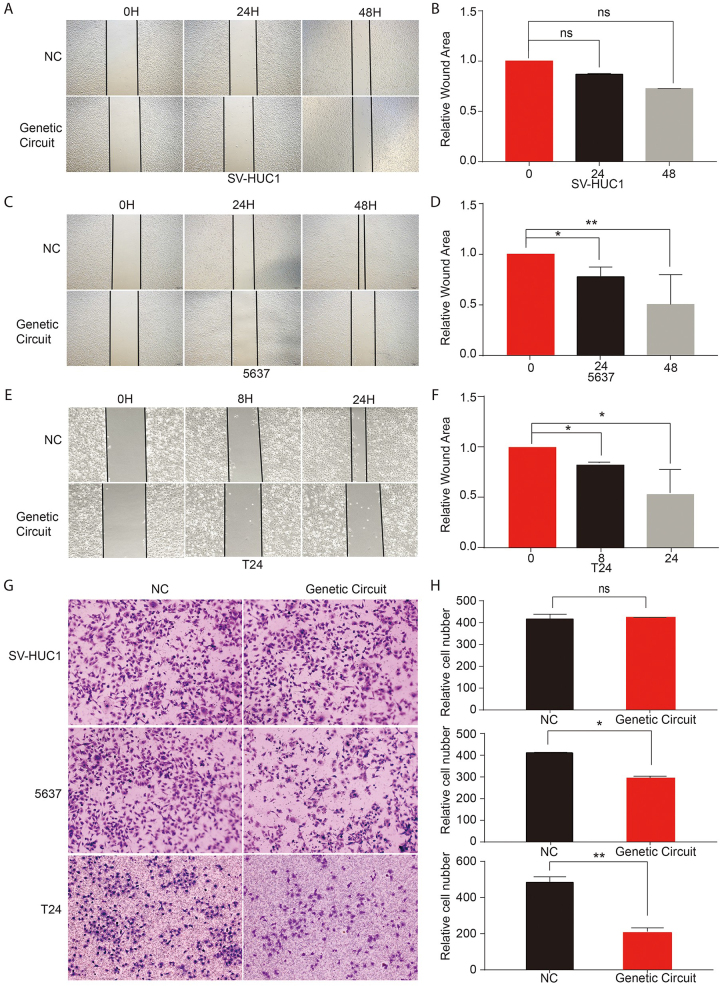
Wound healing assay and Transwell Assays evaluating the effects of the Logic “AND” gate gene circuit on cell migration and invasion. (A, B) SV-HUC1 cells show no significant difference in migration between experimental cells carrying the logic “AND” gate gene circuit and control cells. (C, D) 5637 cells with the logic “AND” gate gene circuit display significantly reduced migration compared to controls. (E, F) T24 cells with the gene circuit also exhibit significantly inhibited migration compared to controls. (G, H) In SV-HUC1 cells, no significant difference in invasion ability is observed between cells with and without the gene circuit. However, in both 5637 and T24 cell lines, the invasion ability is significantly reduced in cells carrying the logic “AND” gate gene circuit compared to the control group. Each experiment was performed at least three times. Statistical significance is indicated as follows: ns (*P* > 0.05), *P < 0.05, **P < 0.01, ***P < 0.001.

We further evaluated the effects of LAG-DTGC on BC cell migration and invasion using scratch and transwell assays. In the scratch assay (Fig. 7A-F), the migration ability of BC cells in the experimental group was significantly reduced compared to the control group (*P* < 0.05), with no significant impact on normal bladder cells (SV-HUC1) (*P* > 0.05). The transwell invasion assay confirmed that invasion capability in the experimental group of BC cells was also significantly inhibited (*P* < 0.05) without affecting SV-HUC1 cells (*P* > 0.05) (Fig. 7G-H). The specific reduction in cancer cell migration and invasion suggests that LAG-DTGC could potentially limit metastasis. By blocking these processes in cancer cells only, LAG-DTGC may reduce the risk of metastatic spread and improve long-term prognosis, as limiting these behaviors is critical for preventing cancer progression and recurrence in patients.

#### LAG-DTGC tool inhibits tumor development and metastasis in vivo

**Figure 8.**
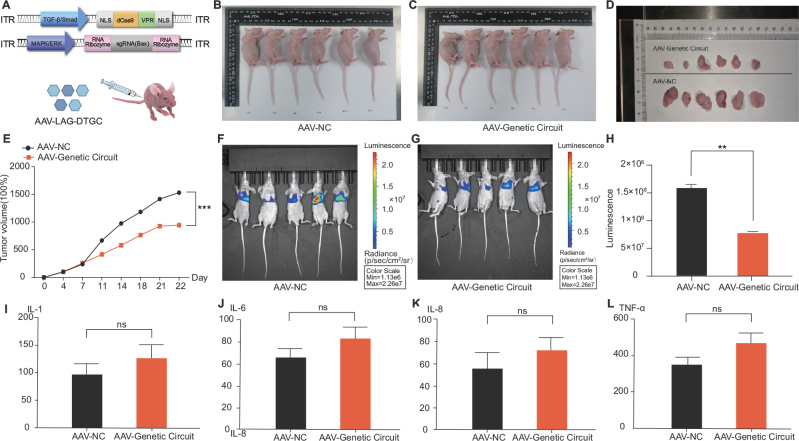
Evaluation of the LAG-DTGC tool in vivo using BALB/c nude mice and B-NDG mice. (A) Schematic representation of the LAG-DTGC tool construction and its delivery into mice via AAV vectors. (B) Images of BALB/c nude mice in the control group (AAV-NC) and the group injected with the AAV-Genetic Circuit (AAV-Genetic Circuit). (C) Images of dissected tumors from BALB/c nude mice in the control group (AAV-NC) and the AAV-Genetic Circuit group (AAV-Genetic Circuit). (D) Comparison of tumor sizes from BALB/c nude mice between the control group (AAV-NC) and the AAV-Genetic Circuit group (AAV-Genetic Circuit). (E) Change in tumor growth over time for B-NDG mice in the control group (AAV-NC) compared to the AAV-Genetic Circuit group (AAV-Genetic Circuit) (***P < 0.001). (F) Luminescence images of B-NDG mice injected with luciferase-expressing bladder cancer T24 cells in the control group (AAV-NC) showing higher tumor burden. (G) Luminescence images of B-NDG mice injected with luciferase-expressing bladder cancer T24 cells in the AAV-Genetic Circuit group showing reduced tumor burden. (H) Quantification of luminescence intensity indicating tumor burden in B-NDG mice. The control group (AAV-NC) shows significantly higher luminescence compared to the AAV-Genetic Circuit group (**P < 0.01). (I-L) ELISA results showing the levels of cytokines IL-1, IL-6, IL-8, and TNF-α in the serum of BALB/c nude mice. No significant differences (ns) were observed between the control group (AAV-NC) and the AAV-Genetic Circuit group for IL-1, IL-6, IL-8, and TNF-α(ns *P* > 0.05, *P < 0.05, **P < 0.01, ***P < 0.001).

To explore the therapeutic potential of the LAG-DTGC tool, we conducted in vivo experiments using both BALB/c nude mice and B-NDG mice models. The LAG-DTGC was delivered via adeno-associated viruses (AAVs), and its efficacy was evaluated in subcutaneous tumor and metastasis models (Fig. 8A). In the subcutaneous tumor model, BALB/c nude mice were injected with 5 × 10^6 T24 BC cells either carrying the LAG-DTGC (AAV-Genetic Circuit) or a non-targeting control (AAV-NC). Tumor volumes were measured twice a week for three weeks. Notably, administration of the LAG-DTGC resulted in a significant reduction in both tumor volume and weight compared to the control group, confirming the effectiveness of the LAG-DTGC in inhibiting tumor growth (Fig. 8B–D). Tumor growth curves (Fig. 8E) further demonstrated a significant decrease in tumor size in the experimental group compared to the control group (***P < 0.001).

To assess the impact of LAG-DTGC on metastasis, we employed a B-NDG mouse model of BC lung metastasis. B-NDG mice were injected with 1 × 10^5^ luciferase-expressing T24 cells via the tail vein, followed by treatment with either AAV-Genetic Circuit or AAV-NC. Luminescence imaging of the mice showed a significant reduction in lung metastases in the LAG-DTGC-treated group compared to the control group (Fig. 8F and G). Quantification of luminescence intensity confirmed the significant decrease in metastatic burden in the experimental group (**P < 0.01, Fig. 8H). Additionally, to evaluate potential inflammatory responses induced by the LAG-DTGC treatment, serum levels of cytokines IL-1, IL-6, IL-8, and TNF-α were measured using ELISA. The results indicated no significant differences in cytokine levels between the LAG-DTGC-treated and control groups, suggesting that the LAG-DTGC does not provoke significant inflammatory responses (Fig. 8I–L, ^ns^*P* > 0.05).

These findings collectively demonstrate the potent anti-tumor and anti-metastatic efficacy of the LAG-DTGC tool in vivo, while maintaining a favorable safety profile by not inducing significant inflammatory responses.

## Discussion

In 2000, Science reported on the synthesis of two artificial gene networks—an oscillator and a switch^[10,11]^, which formally initiated the research known as genetic circuits. The common mode of regulation used in genetic circuits is similar to that of “switches” and “logic gates” in electronic components. “Switches” are molecular structures that can perform specific recognition functions, such as riboswitches and RNA switches. Boolean logic gates are the core of gene circuit design, which can execute Boolean logic functions (NOT, AND, OR). The “AND” gate is a basic digital logic gate that can integrate multiple input signals and only output a signal when all input signals are present^[9]^. For example, Kemmer *et al*^[12]^ designed a gene circuit using an “AND” gate to regulate uric acid homeostasis in vivo. This gene circuit can lower uric acid levels by expressing uric acid oxidase under conditions of high uric acid stimulation. Further experiments showed that when the gene circuit device was implanted into transgenic mice with uric acid oxidase deficiency, uric acid levels were significantly reduced and uric acid crystal deposition in the kidneys was also significantly decreased.

Nowadays, gene circuits, including AND gates, that can sense and process various endogenous signals have been used for cancer identification and treatment^[11,12]^. Although the technique of arbitrary combinations of modular components is well established in gene circuit construction, many previous studies reported regulation of only a few target molecules in the signaling pathway and lacked directional recognition and regulation based on signal networks.

We used to focus on a single molecular effect that promote or inhibit cancer progression. However, signal transduction molecules do not act alone but form networks through interactions during cancer development. “Network vs Network” may be a potentially valuable approach. In our study, a dual-pathway targeting gene circuit was constructed to build a logic AND gate gene circuit in target cells with tumor-specific molecules as recognition signals. CNTN1 and COL14A1 serve as a crucial constituent of LAG-DTGC regulatory networks. By reprogramming oncogenic signaling pathways, LAG-DTGC enables specific intervention in BC cells without affecting normal cells, marking a significant step toward more selective cancer therapies. However, to successfully translate LAG-DTGC into clinical practice, it is essential to consider how various clinical characteristics might influence therapeutic responses. For example, patient age, gender, and TNM stage could significantly impact the efficacy of the LAG-DTGC system. Older patients may exhibit different metabolic profiles and immune responses that affect the effectiveness of the gene circuit. Additionally, the TNM stage can indicate tumor aggressiveness and metastatic potential, which may dictate the intervention strategy. Understanding these clinical characteristics and their interactions with the mechanisms of LAG-DTGC will be vital for optimizing treatment protocols.

Gene editing, high-throughput screening, and bioinformatics technologies continue to evolve, enabling the construction of more sophisticated gene circuits^[13,14]^. Despite these advances, the construction of multi-gene circuits remains technically challenging. It is essential to determine which genes can reliably serve as the basis for these circuits and to understand how their interactions influence therapeutic outcomes. This study suggest that similar expression pattern could be used as a basis for genes screening. Compared with normal cells, CNTN1 and COL14A1 were downregulated in BC cells. It should be noted that whether the artificial gene circuit based on the two genes and signaling pathways can promote apoptosis was unknown before initiation of this study. CNTN1 is a cell surface neuronal adhesion protein that plays a crucial role in the development of neuronal morphology and synapse formation. Studies have shown that the expression of CNTN1 in tumor cells is closely related to the degree of malignancy and prognosis of various tumors, including breast cancer, prostate cancer, colon cancer, ovarian cancer, and lung cancer^[15−17].^COL14A1 is a collagen protein that plays a role in structural support and cell adhesion in the extracellular matrix. Studies have shown that the expression level of COL14A1 is significantly elevated in various tumors, such as breast cancer, colon cancer, lung cancer, and liver cancer, and is closely related to the invasiveness and prognosis of tumors^[18,19]^. Although the role of CNTN1 and COL14A1 in BC cells remain uncertain, but the gene circuit relying on these two genes and their relationship with downstream signaling pathways finally promotes apoptosis. The same trends were also reproduced in in vivo experiments, which showed that the gene circuit inhibited the tumor-forming abilities of BC cells.

BC is the most common malignant tumor in the urogenital system, and precise identification and regulation of BC-specific targets are urgently needed^[13,14]^. During target screening, we used an integrated strategy combining methods such as TCGA-BLCA data, WGCNA, single-factor Cox regression, Lasso regression, and multi-factor Cox regression, ultimately identifying 18 potential targets (e.g., RAD9A, EMP1, NT5DC2, and SLC16A8). This strategy minimizes overfitting and enhances target selection accuracy^[15,16]^.

While these preclinical results are promising, translating this approach to human patients presents several challenges. Human tumors, especially BC, exhibit substantial genetic and microenvironmental heterogeneity, which could influence the specificity and efficiency of LAG-DTGC. Additional studies are needed to optimize circuit components for stability and targeted delivery to maximize efficacy in human cancers. Furthermore, tumor microenvironment factors, such as hypoxia and variable immune responses, could impact circuit function, making it essential to adapt the LAG-DTGC design to accommodate these variables.

As with many targeted therapies, the potential for resistance development poses a challenge, particularly in heterogeneous tumors like BC. Genetic alterations, epigenetic changes, or compensatory signaling pathways could enable tumor cells to evade the LAG-DTGC circuit. For example, mutations in CNTN1 and COL14A1 pathways or alterations in downstream effectors might limit circuit efficacy. Addressing this will require designing circuits with modularity to incorporate multiple signaling checkpoints, potentially making the circuit more adaptable to variations within the tumor microenvironment.

In comparison to other emerging therapies, such as immune checkpoint inhibitors and gene editing technologies like CRISPR-Cas9, the LAG-DTGC system offers a unique advantage by targeting multiple pathways simultaneously. Immune checkpoint inhibitors, though effective in certain cases, are often accompanied by immune-related adverse effects and are not universally applicable due to tumor immune evasion mechanisms. Single-gene therapies, while precise, may fail to address the networked nature of oncogenic signaling in complex tumors. By targeting multiple, tumor-specific pathways, the LAG-DTGC system could provide a more comprehensive approach to managing tumor growth and spread, potentially leading to more durable responses in patients.

The successful construction and testing of LAG-DTGC highlight the potential of synthetic biology in cancer therapy. With further optimization and testing, LAG-DTGC could serve as a foundation for new cancer therapies that are both more precise and less toxic than conventional treatments. This interdisciplinary approach, blending synthetic biology and oncology, demonstrates the potential to develop targeted treatments that are both safe and effective, offering new hope in the fight against cancer.

## Conclusions

In conclusion, our study presents the LAG-DTGC, a targeted gene circuit for BC treatment. The circuit selectively kills cancer cells, demonstrating its potential as a precise therapy. This work highlights the significance of synthetic biology and interdisciplinary collaborations in advancing BC treatment. To successfully translate this approach to clinical applications, future efforts should include safety testing, optimization of delivery systems to enhance targeting and efficacy, and considerations for scaling the system for therapeutic use in human patients.

## Data Availability

All data used in the study were from the publicly available in The Cancer Genome Atlas (TCGA) (https://portal.gdc.cancer.gov/).

## Research registration unique identifying number (UIN)

All data used in the study were from the publicly available The Cancer Genome Atlas (TCGA) (https://portal.gdc.cancer.gov/).
